# Velocity Field Visualization in USP Dissolution Apparatus 3 Using Particle Image Velocimetry

**DOI:** 10.1007/s11095-017-2151-1

**Published:** 2017-04-13

**Authors:** Satish Perivilli, Richard Prevost, Erika Stippler

**Affiliations:** 1 US Pharmacopeial Convention, 12601 Twinbrook Parkway, Rockville, Maryland 20852-1790 USA; 2 LaVision Inc., 211 W. Michigan Ave./Suite 100, Ypsilanti, Michigan 48197 USA

**Keywords:** hydrodynamics, particle image velocimetry, USP dissolution apparatus 3

## Abstract

**Purpose:**

The hydrodynamics in USP dissolution apparatus 3, at five different dip rates, was characterized by analyzing phase-averaged velocity fields obtained using Particle Image Velocimetry (PIV).

**Methods:**

Phase locked 2 Component-PIV (2C–PIV) measurements were recorded on a typical dissolution apparatus 3 configuration with a black painted tablet fixed at the center of the bottom porous screen of the reciprocating cylinder. A trigger mechanism was employed to capture data over 12 phase positions for each reciprocation cycle. Data were captured over a fixed number of cycles, based on dip rate, and the resultant images were post-processed to obtain phase-averaged velocity fields at each phase.

**Results:**

For all dip rates studied, the sinusoidal nature of the cylinder’s reciprocating motion was evident in the images. The phase positions, in which the cylinder was completely submerged, were characterized by recirculation of liquid through the cylinder, top fitting cap, vessel-cylinder annulus, and bottom fitting cap. The direction of recirculation was opposite for phase positions during the up- and downstrokes. The end positions of the up- and downstrokes were characterized by vortices below and above the cylinder respectively. Increasing dip rates led mainly to increasing velocity magnitudes while all flow characteristics, in general, were retained.

**Conclusions:**

The hydrodynamics in typical USP dissolution apparatus 3 is characterized by cyclic phase-dependent flow fields. Specifically, the velocity field distribution within dissolution apparatus 3 is greatly influenced by the relative position of the top cap to the liquid level in the cylinder.

## Introduction

USP dissolution apparatus 3, also known as the reciprocating cylinder apparatus (referred to as App 3 in this paper), is a compendial dissolution apparatus introduced in the *US Pharmacopeia* in 1991 for investigating the performance of modified-release solid oral dosage forms (1). The apparatus typically consists of an array of 6 flat-bottomed cylindrical vessel sets that are used row-wise. The vessels contain dissolution media and are placed in a heated bath. Inner glass cylinders, with porous screens attached at the top and bottom by fitting caps, are allowed to reciprocate in and out of their corresponding vessels at a specific dip rate for a specific time. Evaporation caps fitted at the top of the vessels complete the vessel-cylinder assemblies. At the end of the time allocated for the first row, the cylinders are lifted out and, usually following an additional drain time, moved to subsequent rows. The number of reciprocation cycles in a particular row is defined by the dip rate, measured in dips per minute (dpm), and the time allocated for that row. An upstroke followed by a downstroke constitutes a reciprocation cycle. The dimensions of various components of a vessel-cylinder assembly, along with their allowable tolerances, are specified in USP general chapter *Dissolution* < 711> (2).

Dosage forms are placed on the bottom porous screens of the cylinders. During a reciprocation cycle, the dissolution medium seeps through the porous screens into and out of the cylinder and thus the solutes are carried through a moving medium. In general, the dosage form stays in contact with the bottom porous screen of the cylinder during the upstroke; it may leave the screen and float freely in the cylinder during the downstroke. For this study however, the dosage form was fixed to stay with the bottom porous screen during both the up- and downstrokes of the cylinder.

The influence of geometric and operational parameters on dissolution behavior of various formulations has been reported in literature (3–6). However, scientific literature that focuses exclusively on the hydrodynamics of App 3 is limited (7). This work is an attempt to evaluate the hydrodynamics in a single vessel-cylinder assembly using PIV, an Experimental Fluid Dynamics (EFD) method. Hydrodynamics in a single vessel-cylinder assembly can be assumed to be similar across all such assemblies in a row of the apparatus—provided that there are no geometric or positional variations among physical components.

Various EFD techniques have been used to evaluate hydrodynamics in other dissolution apparatuses. In one of the early works with EFD methods, a Fiber Optics Laser Doppler Anemometer (FOLDA) was used to measure velocity components over different planes along a cross-section of USP dissolution apparatus 2 (App 2) (8). PIV and Laser-Induced Fluorescence (LIF) were also later shown to be suitable tools for providing insight into the hydrodynamics of App 2 (9,10). Additionally, Laser Doppler Velocimetry (LDV) has been used to obtain velocity components at different point locations along the apparatus height (11,12). For mapping the velocity field inside USP dissolution apparatus 4 (App 4), an alternative EFD technique, Magnetic Resonance Imaging (MRI) has been used (13).

Each of the particle-based EFD techniques mentioned above has distinct features and advantages. Single-point methods such as LDV provide a quantitative and accurate sample of the flow at a given point. On the other hand, PIV can provide an instantaneous spatial flow field description (14). Thus, a single PIV experiment can be used to obtain more global data in comparison to LDV, which has to be repeated over multiple regions to generate the same flow structures. For this reason, PIV was chosen in this study to investigate the hydrodynamics in App 3. Literature that explains the concepts of PIV (for example, (14–16)) is readily available. In addition to App 2, this method has also been applied to elucidate flows in stirred tanks (17,18).

## Experimental Method

Phase locked 2C–PIV measurements were taken on a Caleva® Release Rate Tester Type 9 (RRT 9), in which polypropylene screens, of mesh size 20 (defined as standard sieve designation of 850 μm (19)), were used to close both the top and bottom openings of the glass cylinder. A typical non-disintegrating type tablet formulation, coated with black paint to minimize unwanted light reflection from tablet surfaces, was used during PIV data collection. The tablet was fixed at the center of the bottom porous screen using epoxy glue. Any possible effects of tablet size and shape deformation during the test on the apparatus’ hydrodynamics were not considered in this fundamental study. 250 ml of water at 37°C was used as dissolution medium.

### Experimental Setup

**Fig. 1:**
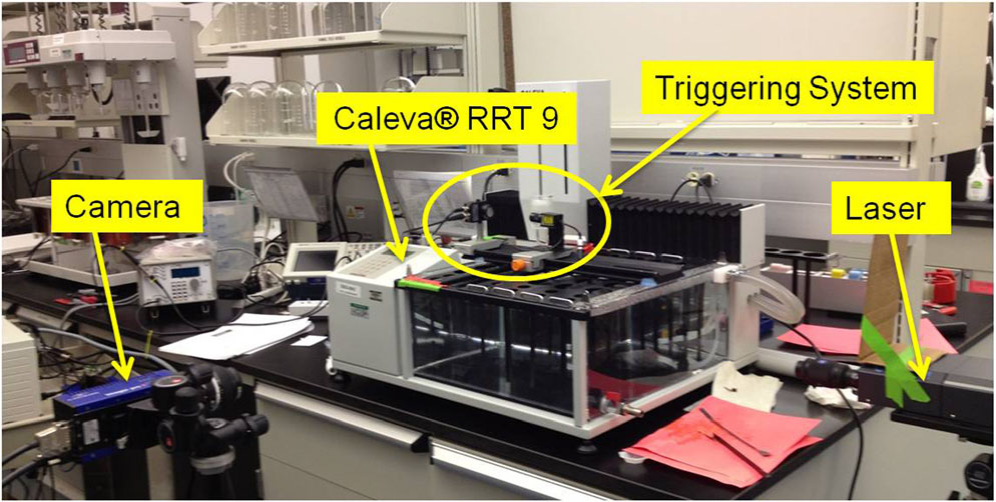
2D-2C PIV system set-up on the Caleva® RRT 9.

PIV data were obtained within a single axial plane of the vessel for all cases. For the measurements, a standard 2 dimensional (2D)-2C PIV system configuration, shown in Fig. 1, was used. A 532 nm laser sheet (provided by a NewWave Solo III 50 mJ dual head laser) illuminated a central axial slice through the vessel/cylinder volume. A charge-coupled device (CCD) camera (LaVision Inc. ImagerProX-2M, 1600x1200 pixel resolution) was used to capture images from the normal of the illuminated plane. The lens (Nikon 50 mm F1.4) and working distance were selected so that the (water) liquid level within the vessel would be imaged over the major axis (1600 pixel) of the camera, thus maximizing the resolution over the observable volume. Fluorescent 3 μm seeding (Thermo Scientific) along with 540 nm high pass camera filters were chosen for this study in order to avoid imaging unwanted reflections from the laser/surface interactions. The seeding is well suited for water application since it is neutrally buoyant at 1.05 g/cm^3^.

### Data Acquisition

PIV measurements were taken at five dip rates: 5 dpm, 6 dpm, 8 dpm, 10 dpm, and 30 dpm. It was initially intended to record data over 32 cycles, composed of two initiating cycles followed by 30 fully developed cycles (i.e, cycles in which the hydrodynamics was completely established) for all dip rates. The selection of 30 cycles for statistical evaluation was to adhere to the large-sample confidence interval requirements in order to provide statistical uncertainty information of the reported mean values. In the course of the measurement campaign it was found that the apparatus could not be operated in a manner that captured the initiating cycles due to triggering limitations (i.e., cylinder reciprocation could not be started from an initial resting position or 0^0^ phase). Therefore, data were collected over 32 fully developed cycles for all runs except those corresponding to 30 dpm. Owing to the speed of the experiment, data were captured over 100 fully developed cycles for the 30 dpm runs.

**Fig. 2:**
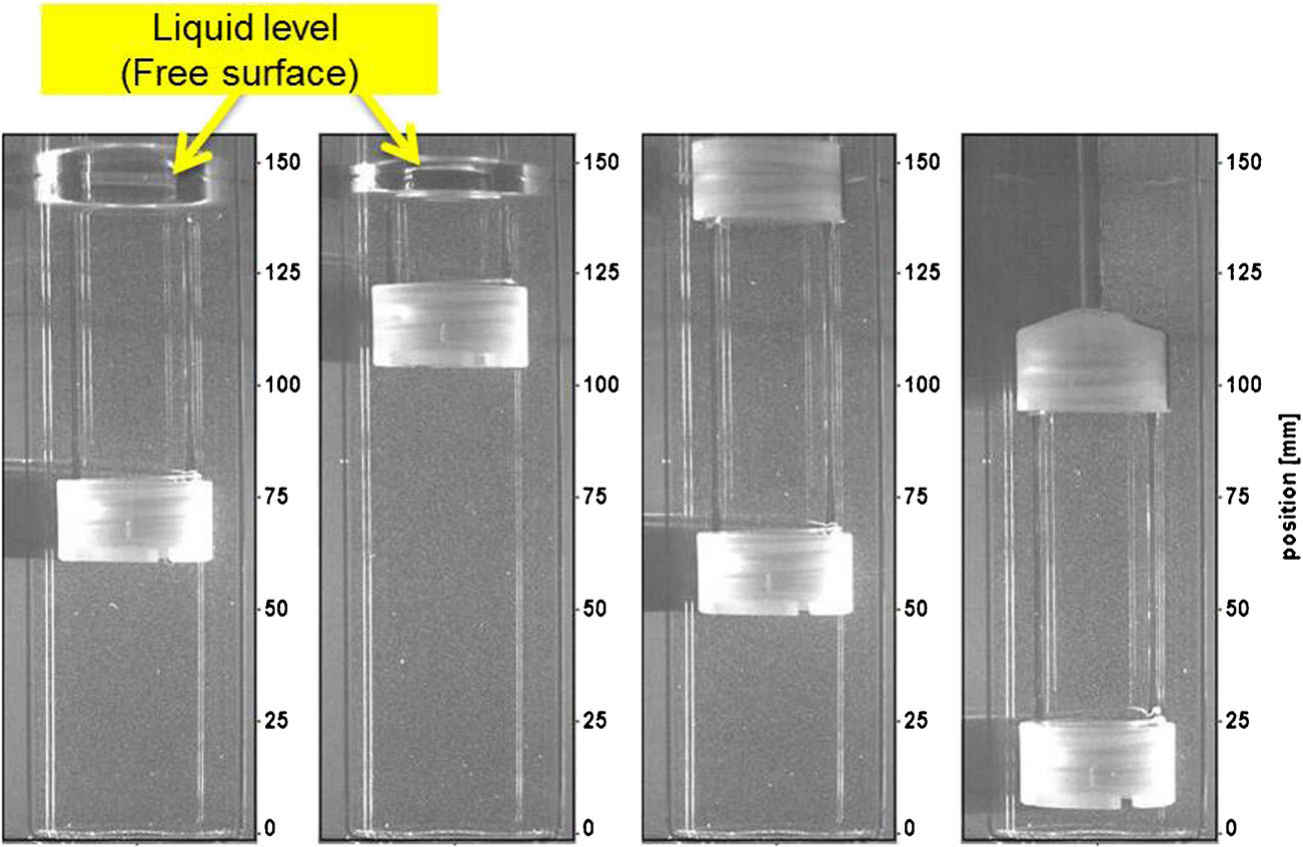
From left to right: Phase Positions 90^0^, 180^0^, 270^0^, and 360^0^ for the 5 dpm case.

For each cycle of cylinder reciprocation, images were acquired at 30^0^ increments for a total of 12 phase positions that varied from 0^0^ to 360^0^ in a reciprocation cycle (from 0^0^ to 180^0^ on the upstroke and from 180^0^ to 360^0^/0^0^ on the downstroke). The aforementioned triggering limitations resulted in an acquisition delay (offset) of 8.6^0^ of the setup for all phase positions. Thus, all references to any phase position (in degrees) should be considered to be in addition to the phase offset (i.e., reported 0^0^ equals actual 8.6^0^, reported 30^0^ equals actual 38.6^0^, and so on). This constant offset, however, propagates into amplified differences in positions at the mid-way points on the up- and downstroke due to the sinusoidal nature of the reciprocation cycle (as illustrated in Fig. 2 for the example case of 5 dpm).

### Data Processing

For each dip rate, the DaVis software (LaVision Inc.) was programmed to capture 12 phase positions (at 30^0^ increments) for 32 cycles (100 cycles for 30 dpm) or a total of 384 images (1200 for 30 dpm) with each image triggered by the incoming stream of cyclic triggers. Every twelfth image of the series was grouped together in a data set resulting in 12 image sets per dip rate (i.e., first set of images at 30^0^, second set of images at 60^0^,. . last set of images at 360^0^). Following this grouping, each of the image sets was post-processed sequentially in the following order:

1. The average of the phase series was subtracted from the instantaneous raw images thereby reducing unwanted reflection in the images. The bright white lines seen in Fig. 2 are examples of reflection in the raw images.
2. All images were then passed through a 3x3 pixel Gaussian filter to improve signal to noise ratio.
3. A pixel intensity value of one count was added to each image to ensure that the image contained no zero counts in any given pixel (and to account for a masking requirement in the vector field calculations).
4. The raw PIV vector field was calculated from the resultant image by using a 32 x 32 pixel interrogation window with a 50% overlap. The algorithm utilized a multi-pass image deformation approach with Gaussian weighted interrogation windows resulting in a measurement resolution of ~3 mm and a spatial resolution of ~1.5 mm. Additional image masking was also used at this step to ensure that the processing only utilized fluid^1^ volume and avoided solid volumes/surfaces (e.g., cylinder/vessel walls, cylinder caps/shaft).
5. A universal outlier detection filter was used to remove erroneous vectors at this stage.
6. The velocity scale was then converted to mm/s for display purposes.
7. Finally, the phase-resolved (average) velocity of the given phase was evaluated from the processed images in this image set. Root Mean Square (RMS) velocities and uncertainties of the average (95% confidence interval) were also evaluated but not reported here for brevity.

The above post-processing procedure was repeated to yield 12 phase-averaged velocity fields (at 12 different phases) for each dip rate.

## Results

**Fig. 3:**
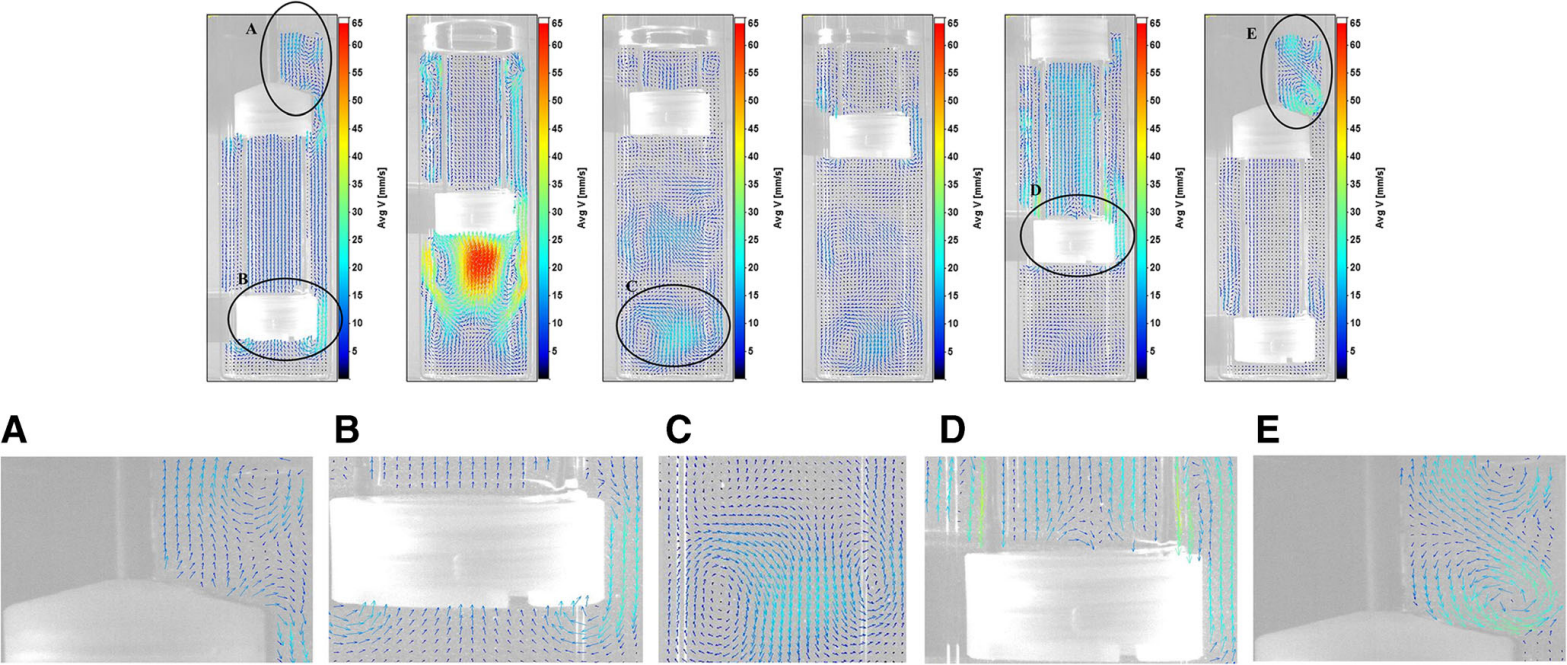
From left to right: Phase-resolved velocity fields for positions 30^0^, 90^0^, 180^0^, 210^0^, 270^0^, and 360^0^ at 5 dpm.

Firstly, the results from the 5 dpm case (treated as baseline results) are presented in detail. Figure 3 shows phase-averaged velocity at six selected phase positions. The sinusoidal nature of the cylinder’s reciprocating motion is evident in the images. Maximum velocity magnitudes were obtained near the cylinder’s mid-way position on the upstroke (90^0^ phase position); minima were observed as the cylinder changed direction towards the end of the upstroke (180^0^ phase).

Closer inspection of the velocity vectors reveals interesting regions at various phases (as illustrated in the magnified regions of selected phases in Fig. 3). As the cylinder moves in its upstroke (for example, phase 30^0^), the surrounding fluid is displaced downward along the annulus of the cylinder and vessel (starting from above the top fitting cap, Region A). The fluid gathers momentum as it passes the bottom fitting cap and produces a recirculation region below the cylinder during the upstroke. The upward momentum of this recirculation region leads to fluid flow through the bottom screen into the cylinder (Region B). This consequently results in fluid flowing upward in the cylinder and out through the air holes in the top fitting cap. A similar recirculation pattern symmetric about the axis of reciprocation was also observed. In addition to this primary recirculation, a secondary vortex was observed above the top fitting cap in Region A. This is caused by the displacement of fluid due to the top cap’s motion, upward fluid motion through the air holes due to primary recirculation, and restrictions from the free surface and the surrounding walls. It can be safely assumed that there would be a similar vortex, symmetric about the shaft, even though the shadow of the shaft prevented data collection. As the cylinder advances in its upstroke to its mid-way position (phase 90^0^), higher velocities were observed in the vessel-cylinder annulus. The velocity in the region below the bottom fitting cap also increases as the fluid replaces the moving solid objects. The primary recirculation and secondary vortex are destroyed by this phase, as the top of the cylinder is past the liquid level by this time. In fact, the secondary vortex was destroyed by phase 60^0^.

After the top cap leaves the free surface on its upstroke, the liquid inside the cylinder was observed to change direction downward by 120^0^. Near the end of the upstroke (phase 180^0^), the velocity magnitudes reach low values and several low intensity vortices appear in regions below the cylinder in the vessel. One of these regions is highlighted and magnified as Region C in Fig. 3. Similar vortices were also found to appear just above Region C. Some weaker vortices were observed within the cylinder.

In the beginning stages of the downstroke (phase 210^0^, for example), the vortices in regions below the cylinder begin to weaken as the cylinder moves downward. By phase 240^0^, the direction of fluid inside the cylinder changed to upward and all vortices in the cylinder were destroyed. By phase 270^0^, representative of the mid-way position on the downstroke, fluid was found to seep through the bottom screen into the cylinder. A recirculation zone in the wake of the tablet was also observed in Region D. Vortices near the vessel bottom were found to persist. It was also found that as the cylinder moves downward, nearby fluid is displaced upward in the vessel-cylinder annulus. As a result, recirculation zones were found to form near the cylinder walls. These flow features are highlighted and magnified as Region D in Fig. 3.

Once the top cap completely re-renters the liquid domain, at phase 300^0^, the liquid inside the cylinder changes direction to downward. Past the mid-way position on the downstroke (i.e., between phases 300^0^ and 360^0^), the primary recirculation (observed earlier between phases 0^0^ and 90^0^) was re-established—but in the opposite direction. Near the end of the downstroke (phase 360^0^), velocity magnitudes were reduced considerably and existent vortices were observed to weaken. A comparatively stronger set of vortices, energized by the upward momentum of flow in the vessel-cylinder annulus, were found to develop in the region above the top cap (Region E). As the cylinder returns to its upstroke, the energy in these vortices begins to dissipate as shown in Region A at phase 30^0^.

**Fig. 4:**
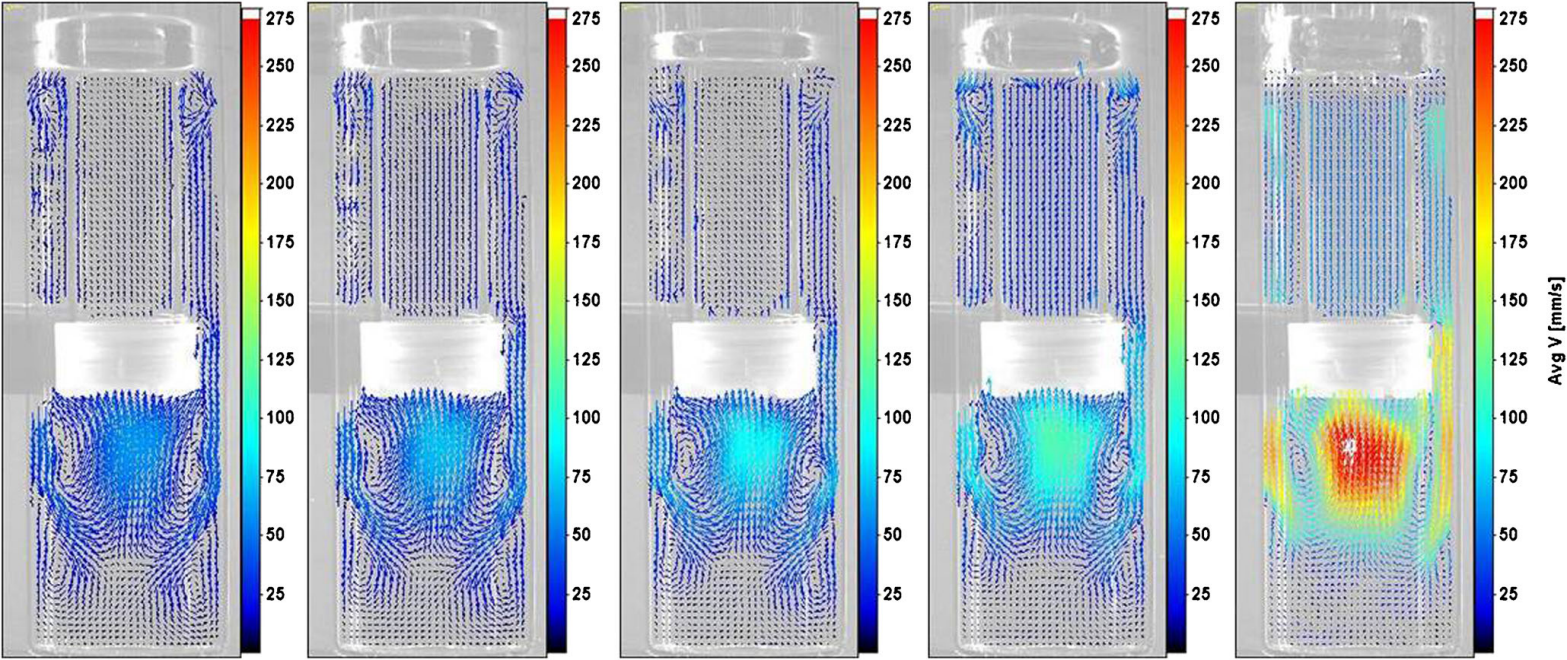
From left to right: Velocity fields at 5 dpm, 6 dpm, 8 dpm, 10 dpm, and 30 dpm at phase 90^0^.

The phase-averaged velocity fields at 5 dpm were compared with results across other dip rates. On the upstroke, the primary recirculation with liquid inside the cylinder flowing upward (and out through the air holes in the top cap) and downward through the vessel-cylinder annulus was observed at all dip rates between phases 30^0^ and 60^0^. At phase 30^0^, secondary vortices similar to those seen above the top cap at 5 dpm were also observed at other dip rates. These vortices were destroyed by phase 60^0^ for all dip rates. Similarly, at phase 90^0^ (shown in Fig. 4), the primary recirculation was destroyed as the top cap leaves the free surface and liquid inside the cylinder changed direction to downward at phase 120^0^ over all dip rates. In general, the flow behavior was also retained across dip rates between phases 120^0^ and 150^0 0^.

**Fig. 5:**
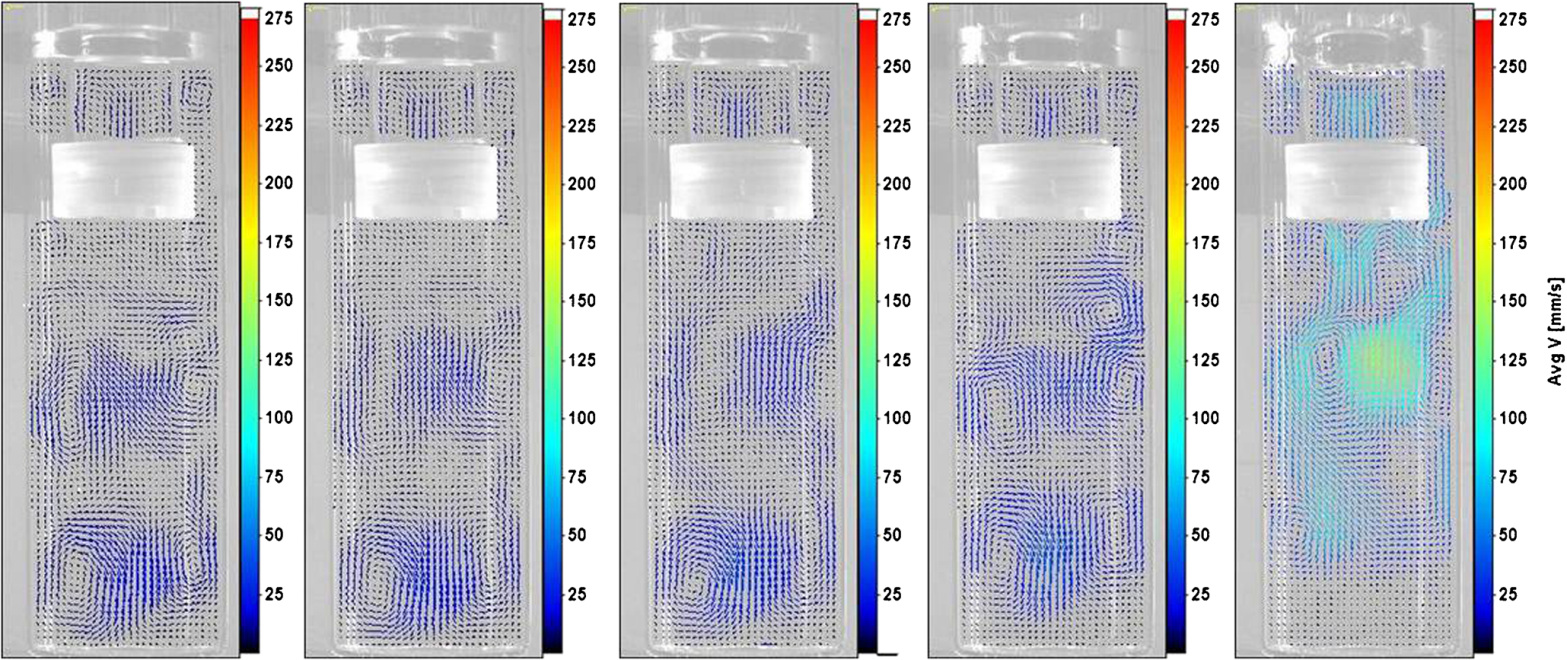
From left to right: Velocity fields at 5 dpm, 6 dpm, 8 dpm, 10 dpm, and 30 dpm at phase 180^0^.

**Fig. 6:**
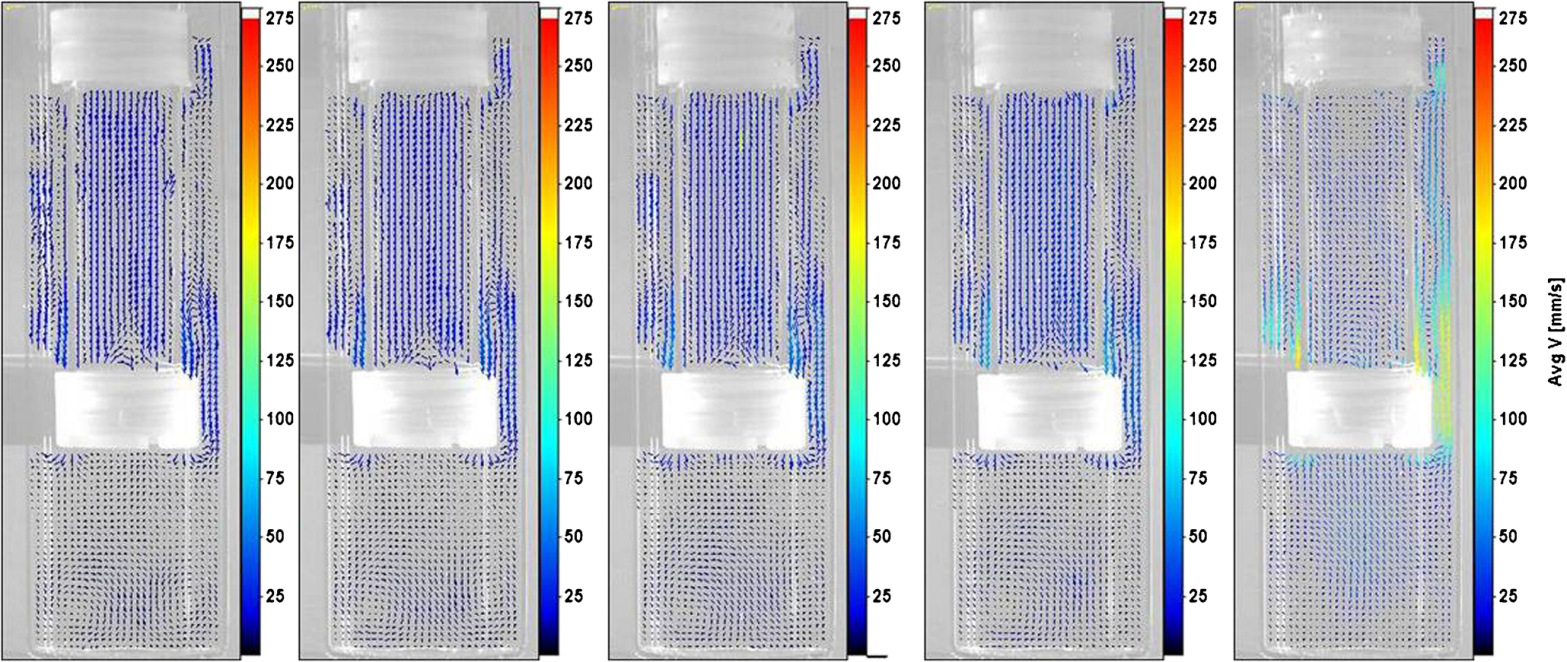
From left to right: Velocity fields at 5 dpm, 6 dpm, 8 dpm, 10 dpm, and 30 dpm at phase 270^0^.

Some of the vortices formed below the cylinder in the vessel at phase 180^0^ were not found at 30 dpm even though the essential flow features remained the same with increasing dip rates (as shown in Fig. 5). On the downstroke, the weak vortices within the cylinder, between phases 180^0^ and 210^0^ at 5 dpm, were found to be retained for dip rates up to 10 dpm. However, for the 30 dpm case, these vortices were smaller and flow inside the cylinder was found to be predominantly downward from phases 180^0^ to 240^0^. Also, the complete change of direction of flow inside the cylinder to upward was observed to be slightly delayed for the 30 dpm case. The direction change occurred at phase position 240^0^ for 6 dpm to 10 dpm, and at 270^0^ for 30 dpm. However, flow features were observed to be similar once the direction transition occurred with increasing dip rates. The comparison of velocity fields across all dip rates at phase 270^0^, the mid-way position on the downstroke, is shown in Fig. 6.

**Fig. 7:**
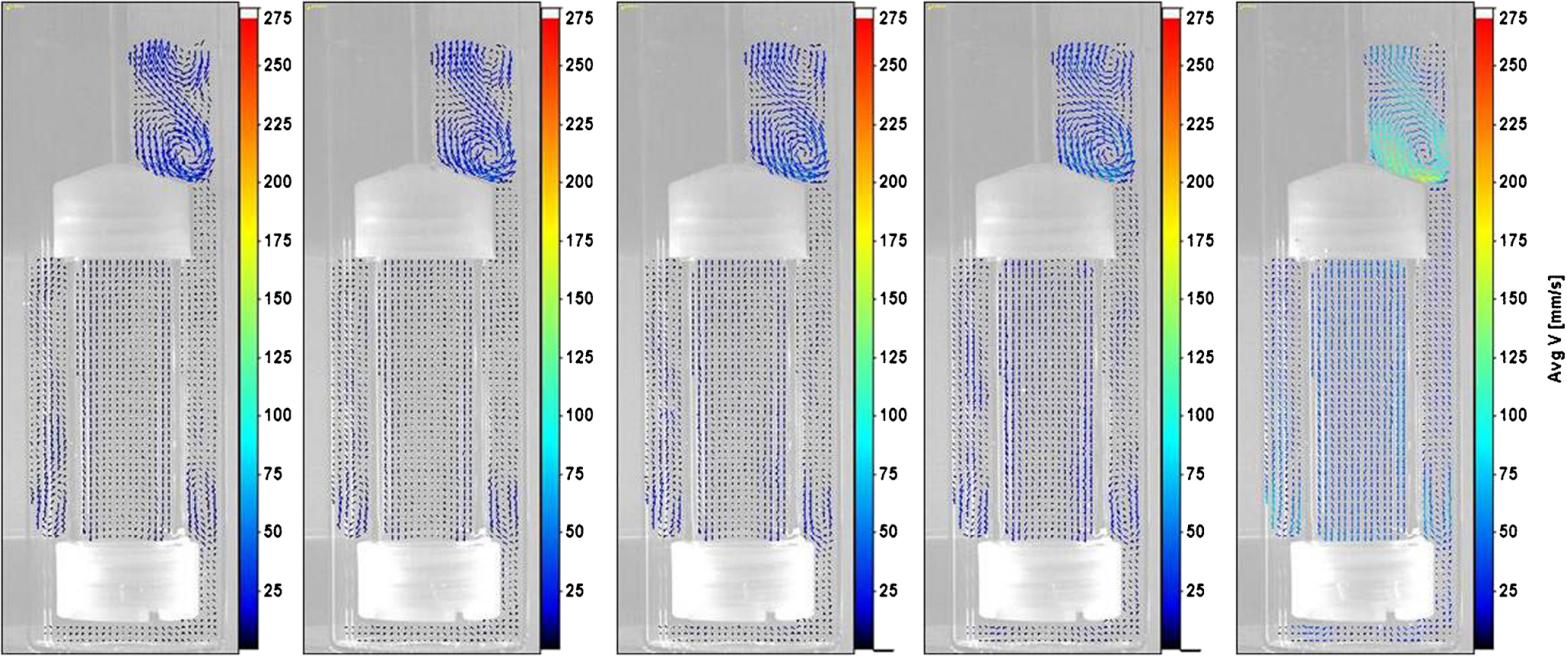
From left to right: Velocity fields at 5 dpm, 6 dpm, 8 dpm, 10 dpm, and 30 dpm at phase 360^0^.

By phase position 300^0^, the direction of liquid within the cylinder was downward for all dip rates. Between phase positions 300^0^ and 360^0^, the recirculation zone observed at 5 dpm was also established over the other dip rates considered. In addition, the stronger set of vortices above the top cap at phase 360^0^ was also found at the higher dip rates considered (as shown in Fig. 7).

Thus, in general, the velocity vectors showed qualitative similarity (albeit with increasing magnitude) as the dip rate increased from 5 dpm to 30 dpm at all phase positions with certain exceptions at 30 dpm.

## Discussion

Hydrodynamic conditions define the velocity gradients/stresses that a dosage form experiences in a dissolution apparatus. Thus, understanding the hydrodynamics involved in the operation of an apparatus is a critical step toward gaining insights into operational parameters which could influence the dissolution behavior of a dosage form. EFD methods have previously been used to investigate the hydrodynamics in different dissolution apparatuses (8–13). However, there are no readily known published efforts to date that describe the hydrodynamics in App 3 using EFD methods. For this study, PIV was used due to its distinct advantage of providing instantaneous full field flow data. Velocity fields similar to those presented in (9,10,17,18) were generated for App 3 using phase locked 2C PIV. Results were obtained at five different dip rates.

Irrespective of the dip rate, position of the top cap relative to the free surface (liquid level) influences the direction of liquid within the cylinder. On the upstroke, the flow direction changes from upward to downward as the top cap leaves the free surfaces between phases 90^0^ and 120^0^. The motion of the top cap past the free surface was also found to destroy the secondary vortices formed above the top cap. On the downstroke, the flow direction changes from downward to upward as the top cap re-enters the liquid domain between 240^0^ and 270^0^ for all dip rates.

Between phases 150^0^ and 210^0^, when the top cap is completely above the liquid level, vortices were observed below the cylinder. Also, weak vortices were found to form inside the cylinder with the exception of the 30 dpm case between phases 180^0^ and 210^0^. For the 30 dpm case, these vortices were not as prevalent, owing to the higher agitation rate, and the liquid was directed predominantly downward for all phase positions between 120^0^ and 240^0^.

When the cylinder is completely submerged in the liquid (i.e., between phases 0^0^ and 60^0^ on the upstroke and 300^0^–360^0^ on the downstroke), the flow in App 3 is characterized by primary recirculation patterns involving motion of liquid within the cylinder, the air holes in the top cap, and the vessel-cylinder annulus. A key observation, however, is that the recirculation patterns are directed opposite to each other on the up- and downstrokes. At phase 360^0^, vortices that are energized by the upward momentum of fluid in the vessel-cylinder annulus were found in the region above the top cap.

## Conclusions

PIV was successfully used to capture the hydrodynamics in App 3 at five different dip rates. Irrespective of the dip rate, the cyclic nature of the fluid flow was evident from all PIV measurements taken. Also, global velocity magnitudes were observed to be at a maximum when the cylinder was mid-way on the upstroke and minimum towards the end of the upstroke for all dip rates considered.

Velocity fields at different phases of interest were analyzed. Over all dip rates, a primary recirculation in which the liquid moves upward inside the cylinder and through the top porous screen (and air holes), back down and through the vessel-cylinder annulus, and then upward towards the bottom porous screen, was observed when the cylinder is completely submerged on the upstroke. Similar recirculation, but opposite in direction, was observed when the cylinder was completely submerged during the downstroke. Also, on the upstroke, vortices were created and energized below the cylinder; on the downstroke, vortices were created and energized above the cylinder. The ends of stroke positions were characterized by weakening of the existent vortices. However, a strong set of vortices were found to develop above the top fitting cap by the end of the downstroke.

In conclusion, the hydrodynamics in App 3 is characterized by distinct phase-dependent flow features. Increasing dip rates led mainly to increasing velocity magnitudes in the flow domain, while most of the flow characteristics were retained with the exception of velocity fields at certain phase positions at the highest dip rate considered.

## Abbreviations

2C: 2 Component
2D: 2 Dimensional
App 2: USP dissolution apparatus 2
App 3: USP dissolution apparatus 3, also known as the reciprocating cylinder apparatus
CCD: Charge-coupled device
dpm: dips per minute
EFD: Experimental Fluid Dynamics
FOLDA: Fiber Optics Laser Doppler Anemometer
LDV: Laser Doppler Velocimetry
LIF: Laser-Induced Fluorescence
MRI: Magnetic Resonance Imaging
PIV: Particle Image Velocimetry
RRT 9: Release Rate Tester Type 9

## Footnotes

1 The term fluid in this article refers to liquid (water) in the apparatus.
